# Physiological Perturbation Reveals Modularity of Eyespot Development in the Painted Lady Butterfly, *Vanessa cardui*

**DOI:** 10.1371/journal.pone.0161745

**Published:** 2016-08-25

**Authors:** Heidi Connahs, Turk Rhen, Rebecca B. Simmons

**Affiliations:** 1 Biology Department, University of North Dakota, Grand Forks, North Dakota, United States of America; 2 Department of Biological Sciences, National University of Singapore, Singapore; Monash University, AUSTRALIA

## Abstract

Butterfly eyespots are complex morphological traits that can vary in size, shape and color composition even on the same wing surface. Homology among eyespots suggests they share a common developmental basis and function as an integrated unit in response to selection. Despite strong evidence of genetic integration, eyespots can also exhibit modularity or plasticity, indicating an underlying flexibility in pattern development. The extent to which particular eyespots or eyespot color elements exhibit modularity or integration is poorly understood, particularly following exposure to novel conditions. We used perturbation experiments to explore phenotypic correlations among different eyespots and their color elements on the ventral hindwing of *V*. *cardui*. Specifically, we identified which eyespots and eyespot features are most sensitive to perturbation by heat shock and injection of heparin—a cold shock mimic. For both treatments, the two central eyespots (3 + 4) were most affected by the experimental perturbations, whereas the outer eyespot border was more resistant to modification than the interior color elements. Overall, the individual color elements displayed a similar response to heat shock across all eyespots, but varied in their response to each other. Graphical modeling also revealed that although eyespots differ morphologically, regulation of eyespot size and colored elements appear to be largely integrated across the wing. Patterns of integration, however, were disrupted following heat shock, revealing that the strength of integration varies across the wing and is strongest between the two central eyespots. These findings support previous observations that document coupling between eyespots 3 + 4 in other nymphalid butterflies.

## Introduction

Eyespots are one of the most striking and diverse features displayed on butterfly wings. These colorful pattern elements are composed of concentric rings or pattern elements that can vary widely in size, number and color composition even on the same wing surface. The contrasting colors of concentric rings create a bold, conspicuous pattern, which may have evolved as a visual signal to intimidate or deflect predators [1–3]. In some butterfly species, eyespots appear to have evolved specialized functions; dorsal eyespots are employed for courtship display while ventral eyespots are used for predator deterrence [4,5]. The complexity and diversity of butterfly eyespots has drawn the attention of evolutionary biologists that seek to understand not only their functional role, but also the underlying developmental program generating these patterns [6–11].

Several models have been proposed to explain eyespot formation based on manipulations of eyespot development and identification of genes involved in initial eyespot establishment [11–15]. Eyespot specification begins in the late larval wing discs where a group of organizing cells, the focus, form the presumptive eyespot center [16,17]. Gradient models propose that organizing cells emit one or more putative long-range morphogens that diffuse radially, forming a concentration gradient [12,18]. During early pupation, the surrounding epidermal scale cells are thought to respond to positional information specified by the concentration gradient. These scale cells trigger an unknown series of molecular events that lead to the synthesis of different colored pigments. Although the identity of the focal signaling molecule(s) is currently unknown, a number of transcription factors and morphogens have been implicated in regulating eyespot development [13,19]. Interestingly, the genes identified in eyespot development are the same as those involved in wing development; thus, co-option of the wing gene regulatory network may explain how eyespots originated [20]. Experimental data suggests that modifications of developmental networks may have generated eyespot diversity by altering properties of the focal signal and/or response thresholds of scale cells [7]. As an alternative, the induction model proposes that eyespot development and diversity arises from variation in the speed, timing and duration of multiple signals released from the eyespot focus [15,21]. These signals are believed to be self-enhancing at short range, triggering development of dark rings, while light rings represent gaps formed by long-range inhibitory signals [21]. A recent comparative analysis of these models argues that the gradient model is better supported by experimental data [19].

In many butterflies, eyespots develop as a series of homologous pattern elements along the wing margin known as the border ocelli system [22]. Each eyespot develops within a wing cell created by a border of wing veins that mark different wing compartments. Artificial selection for eyespot size, color and shape have revealed correlations among serially repeated eyespots, providing strong evidence of developmental integration [12,23–25]. Genetic coupling among eyespots, perhaps due to linkage or pleiotropy, prompted the idea that the entire group of ocelli functions as a discrete integrated unit separate from other pattern elements on the wing [26,27]. Eyespots also appear to exhibit integration in response to experimental perturbation where they are coordinately dislocated either proximally or distally on the wing [28,29].

Despite evidence of developmental integration among eyespots, many studies have also documented modularity (individuality) between wing compartments. Selection experiments demonstrated that it is possible to uncouple the size of eyespots on the same wing surface [7,12,30]. These results suggest that eyespots in different wing cells are regulated independently either by different networks, sub-networks or differences in network sensitivity to regulatory molecules. Investigation of asymmetry within eyespots across diverse butterfly species indicates that individual colored rings may also develop independently of each other [15].

Modularity is a common theme in organismal development, enabling spatial partitioning of semi- autonomous developmental units that are then free to diversify in function or morphology [31–33]. In this way, modularity can promote flexibility during development [33]. Modularity may facilitate rapid responses to environmental heterogeneity through phenotypic plasticity by permitting independent networks or sub-networks to be induced by environmental cues [34–36]. This idea is pertinent to eyespot development as many butterflies exhibit phenotypic plasticity in response to changing environmental conditions with some eyespots exhibiting greater sensitivity than others [7,37,38].

To better understand the evolution of eyespot diversity, researchers have used a variety of experimental approaches to modify eyespot development. The most common approach is to conduct perturbation experiments (cautery, temperature shock, injection of hormones and pharmacological agents e.g. heparin, sodium tungstate and thapsagargin) and examine which aspects of pattern development are modified [37,39–42]. Although modifications are not always representative of phenotypic plasticity in wild populations, some of these modifications mimic patterns found in related species or resemble aberrant wing patterns occasionally observed in nature [40,43]. Studying which eyespots or eyespot features are more susceptible to modification may reveal developmental biases or constraints that have influenced the evolution of eyespot diversity [26,40].

Here we explore patterns of integration and modularity in the globally distributed butterfly, *Vanessa cardui* (Nymphalidae) by investigating how individual eyespots and their corresponding color elements respond to two different physiological perturbations, heat shock and heparin. We were interested in identifying whether eyespots are highly integrated or whether certain eyespots, combinations of eyespots, or eyespot color elements are susceptible to perturbation, thus indicating modularity in development. Previous work in *V*. *cardui* has shown that temperature shock during early pupation can alter wing patterns [29,43] with cold shock inducing dramatic changes in eyespot development [40]. Interestingly, injection of pharmacological agents such as heparin precisely mimic the effect of cold shock on wing patterns including eyespots [39,44,45]. Heat shock can also induce wing pattern changes in *V*. *cardui*; however, the effects are often subtle [28] and it is unclear whether the eyespots are affected. Here, we show that eyespots are affected by heat shock. Although heat shock and heparin induce dramatically different effects on eyespot development, some common patterns emerge regarding which eyespots and color elements are most strongly or weakly affected.

## Materials and Methods

### Butterfly Rearing and Experimental Setup

*Vanessa cardui* caterpillars and artificial diet were purchased from Carolina Biological Supply Company (carolina.com). Caterpillars arrived as 2^nd^ instar larvae and were randomly assigned to the following treatment groups: heat shock, heparin injection or control (unmanipulated). Caterpillars were reared in individual containers in ambient conditions (23°C) under a 12-hour light/dark cycle, and were fed 3 grams of artificial diet every other day until pupation at approximately 7 days. Within 12 hours of pupation caterpillars assigned to the heat shock treatment were transferred to an incubator set at 37°C for 48 hours. Caterpillars assigned to the heparin group were injected with 2 μl (10 μg) of heparin (Sigma Aldrich) using a Hamilton syringe (2mm) within 12 hours of pupation [39,46]. Injections were performed at the margin of the left wing and the needle was cleaned with 70% ethanol between each injection. Sham injections were also performed with and without water for a small group of pupae to ensure there was no effect of the needle or water injection on the wing phenotype. Following the treatments, all pupae were returned to the same rearing conditions as the control group. All pupae were placed in clean containers with paper tissue to act as support material to ensure successful eclosion of butterflies. Throughout the experiment, containers containing caterpillars and pupae were arranged with alternating treatment groups to reduce potential variation in environmental conditions.

### Eyespot Color Analysis

Following eclosion, butterflies were immediately placed at -20°C for storage until wing pattern analysis. Butterflies were spread on a pinning board for a minimum of 3 days. Hindwings were carefully removed and images were captured using a Canon digital camera (DP70) mounted on an Olympus SZX12 microscope. Images of the ventral surface included a scale bar. Morphometric measurements of eyespots were made using ImageJ (imagej.nih.gov/ij) with the number of pixels calibrated to one cm. The position of eyespots were identified by reference to the nearest adjacent vein; we examined eyespots 2, 3, 4 and 5 in wing cell compartments M2, M3, Cu1 and Cu2 respectively [47,48]. Eyespots are abbreviated as ‘ES’ in figures and tables.

**Fig 1:**
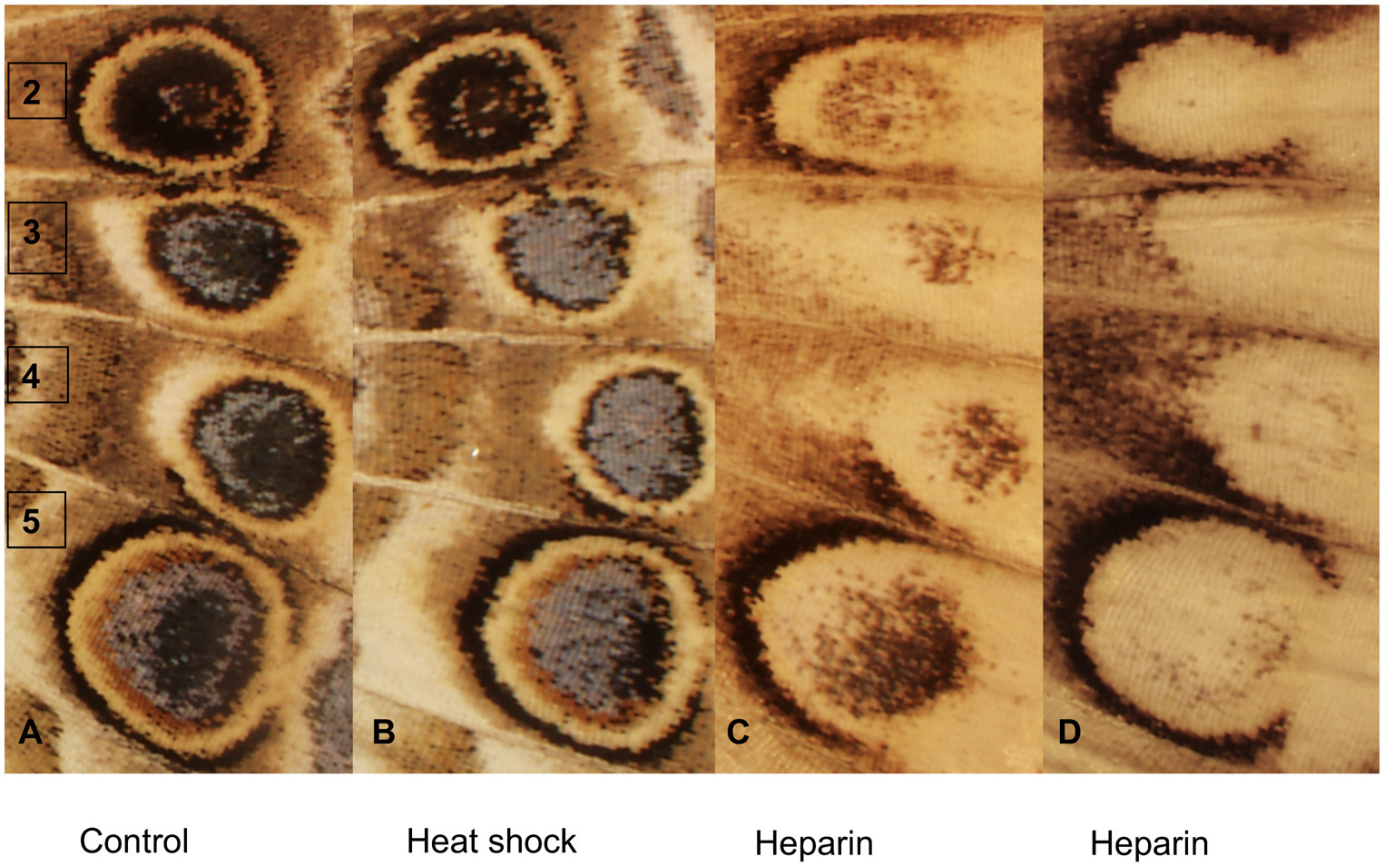
Images of eyespots from the different treatment groups illustrating representative phenotypes. **Panel A** Control (un-manipulated), **Panel B** Heat shock, **Panel C** Heparin, **Panel D** Heparin extreme phenotype with complete loss of central eyespots. (2–5 = Eyespots 2–5).

**Fig 2:**
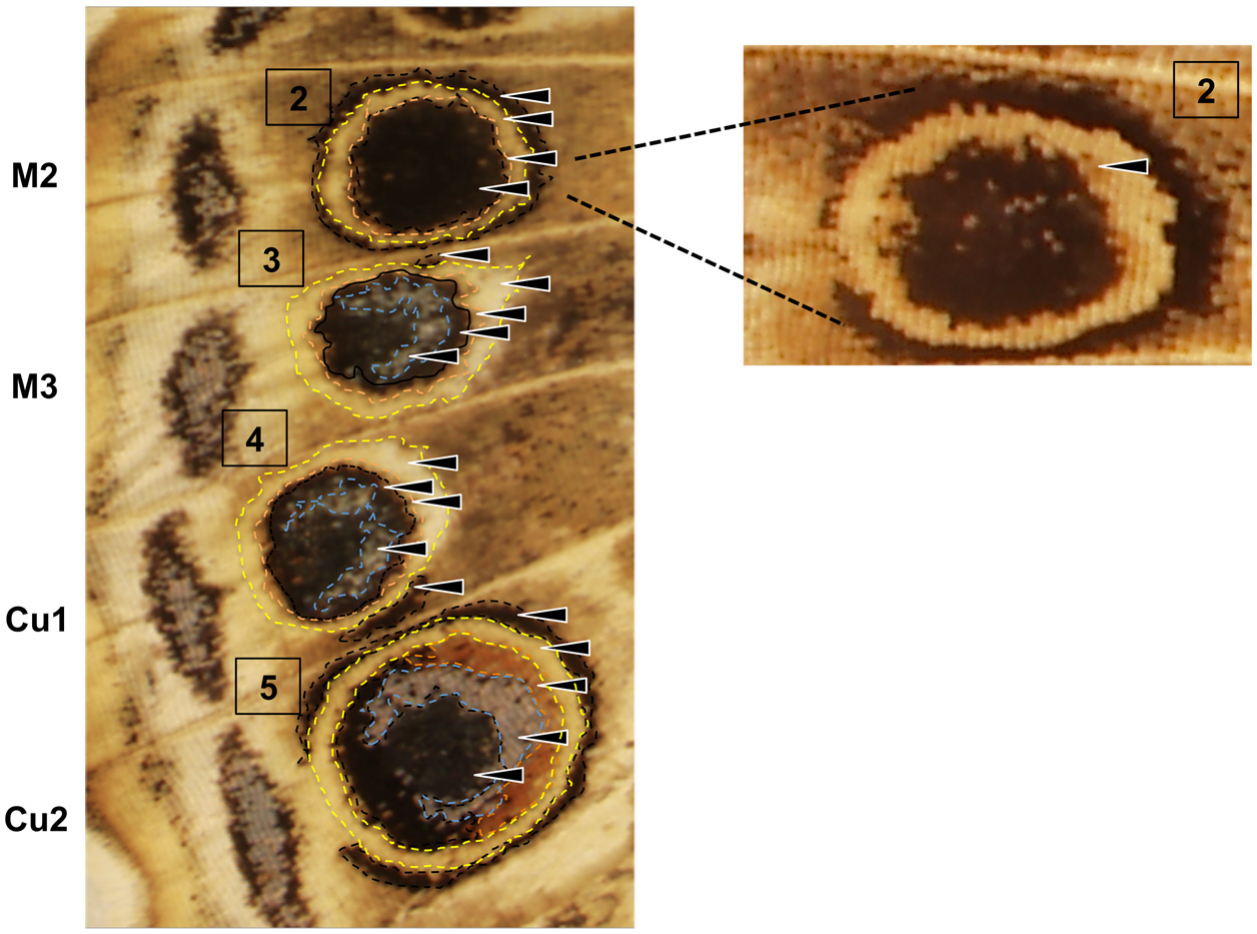
Color composition of eyespots in *Vanessa cardui*. Each of the four eyespots represents a combination of these different color elements that were measured for the color pattern analysis. Blue scales are rarely observed in the center of eyespot 2 and therefore were not measured for this eyespot. Dashed lines approximate the areas of each color measured in Image J. Arrows point to each of the five pattern elements measured. Close up of eyespot 2 shows orange pigment around the black focus.

*V*. *cardui* have a series of marginal eyespots composed of different colors that approximate five concentric rings; an outer black border, a yellow, an orange and a blue region, and finally a black center (Figs 1 and 2). These individual colors do not occur as clearly defined rings as observed in some other butterfly species e.g. *Bicyclus anynana* but develop either as fragmented rings or different colored regions within a circular pattern element. For example, the black border is reasonably defined as a ring in eyespots 2 and 5, however only a small fragment of black pigmentation is observed in the border region of the two central eyespots, which we assume to be homologous to the black border of eyespots 2 and 5. Each color is assumed to be pigment likely derived from the synthesis of either melanin or ommochrome pathways with the exception of blue, which is a structural color. For simplicity we refer to the different colored components of eyespots as color elements. Measurements of each color element and eyespot area were measured in ImageJ as illustrated in Fig 2. Wing size was also measured by carefully drawing around the edge of the wing using ImageJ. All wings and color elements were each measured a minimum of two times to ensure accuracy.

Total eyespot area and sum of the area of the different color elements were compared throughout the process to ensure measurements were consistent. The heat shock experiment was replicated three times and the heparin experiment was conducted once because preliminary studies indicated that heparin had dramatic effects on wing patterns. Heparin eyespots were measured twice and the average of these measurements was used in the final analyses.

### Statistical Analyses

Measurements of total eyespot area and sum of eyespot color elements were assessed using bivariate correlations to ensure high repeatability between the two sets of measurements for each eyespot. A two-way ANCOVA was performed using Type III sums of squares to compare differences in eyespot size and area of the different colored pattern elements both within the control group and also between the control and treatment groups (Number of individuals: Control *n* = 45, Heparin *n* = 20, Heat shock *n* = 46). The model consisted of treatment and eyespot as main effects and overall wing size as a covariate for eyespot size. Batch was included as a blocking factor for replicated experiments (batch x treatment) and interaction terms were examined for treatment x eyespot and wing x treatment where appropriate. All data were examined for normality of residuals (Shapiro Wilk p>0.05) and equal variance (Levene Test p>0.05). A Dunn-Šidák correction for multiple comparisons was used when the interaction term was significant. All analyses were performed in JMP version 11 (SAS, Institute, Cary, NC). The heparin treatment caused a large number of outliers for the whole model. Thus, heparin was excluded from some of these analyses due to the presence of many zero measurements.

Associations among eyespots and eyespot traits were assessed using measures of conditional independence (modularity) and graphical modeling [32]. Graphical Modeling was conducted for eyespots from the control and heat shock treatment only. Partial correlations between all eyespots for eyespot size and percent area of each eyespot element were obtained for the control and heat shock treatments separately using multiple regression after checking for normality and equal variances between all associations (Control *n* = 45, Heat shock *n* = 46). Wing size was included as a covariate. Partial correlations represent the association between two variables (size or color) after accounting for correlations among all other variables. Edge Exclusion Deviance (EED) is a theoretical measure of whether a particular edge can be eliminated from a saturated model of complete integration [32]. EED was calculated using the following formula–N ln [1-pij^2^] where N represents sample size and pij is the partial correlation between two variables. The strength of the edge (correlation) was calculated using–(0.5) ln [1-pij^2^]. The value of each EED is tested against the χ^2^ distribution with one degree of freedom. Values less than 3.84 (p<0.05) are rejected as having an edge (i.e. the traits are not significantly integrated thus inferring modularity) [33]. Values greater than 3.84 indicate the traits are developmentally integrated (not conditionally independent). The matrix of EED values was used to construct a graphical model illustrating patterns of integration and modularity among all eyespots. Eyespots that are not connected (i.e. those with no edge) are inferred to be conditionally independent (modular). Integrated eyespots are those with significant edges that are correlated independent of their associations with the other eyespots.

### Ethics Statement

This research did not require any permits to obtain the butterflies and did not involve any endangered or protected species.

## Results

### Bivariate analyses and wing area measurements

All results for multiple comparisons using the Dunn-Šidák post hoc test are presented in Table 1. Bivariate plots for regression analyses of total eyespot area and sum of pigment area revealed a high correlation between these two sets of measurements for all eyespots across each treatment (r^2^ = 0.95–0.99) (S2 Fig). A one-way ANCOVA was conducted to compare wing area across the treatment groups using batch as a covariate. There was no batch by treatment interaction for the replicated experiments (F _(2, 313)_ = 1.9, p<0.14) and no effect of either treatment on wing area (F _(2, 92)_ = 2.8, p = 0.07). As wing area was not significantly different between treatments, it was used as a covariate in a 2-way ANCOVA to examine differences in eyespot size. The sham injections had no effect on any trait examined (data not shown).

**Table 1:** Dunn-Šidák corrections for multiple comparisons following 2-way ANCOVA for treatment x eyespot interactions comparing changes in colored eyespot elements (cm^2^). Significance is indicated when p<0.006 for 14 comparisons and p<0.009 for 6 comparisons. (ES refers to eyespot).

Eyespot multiple Comparisons	Eyespot Area	Black border	Yellow	Orange	Blue	Black focus
Control ES5 vs. ES4	p<0.0001	p<0.0001	NS	p<0.0001	p<0.0001	NS
Control ES5 vs. ES3	p<0.0001	p<0.0001	p<0.0002	p<0.0001	p<0.0001	p<0.0001
Control ES5 vs. ES2	p<0.0001	p<0.0001	p<0.0001	p<0.0001		p<0.001
Control ES3vs. ES4	p<0.0001	NS	p<0.05	NS	NS	p<0.0001
Control ES2 vs. ES4	NS	p<0.0001	p<0.0001	NS		p<0.0001
Control ES2 vs. ES3	NS	p<0.0001	p<0.0001	p<0.008		p<0.0001
Control ES5 vs. Heparin ES5	NS	NS	NS	NS		p<0.005
Control ES5 vs. Heat shock ES5	p<0.001	NS	p<0.006	NS	NS	p<0.0001
Control ES4 vs. Heparin ES4	p<0.0001	NS		p<0.0001		p<0.0001
Control ES4 vs. Heat shock ES4	p<0.01	p<0.05	p<0.0001	NS	p<0.0001	p<0.0001
Control ES3 vs. Heparin ES3	p<0.0001	p<0.0001		p<0.0001		p<0.0001
Control ES3 vs. Heat shock ES3	p<0.002	NS	p<0.0001	NS	p<0.005	p<0.0001
Control ES2 vs. Heparin ES2	NS	p<0.05	p>0.05	p<0.0001		p<0.0001
Control ES2 vs. Heat shock ES2	NS	NS	NS	NS		p<0.0001

Only 6 comparisons shown for the blue element due to absence of blue in eyespots of butterflies treated with heparin and the lack of blue in eyespot 2 for all treatment groups. Yellow pigment was not measured in ES3 and ES4 for the heparin treatment.

### Eyespot Size

**Fig 3:**
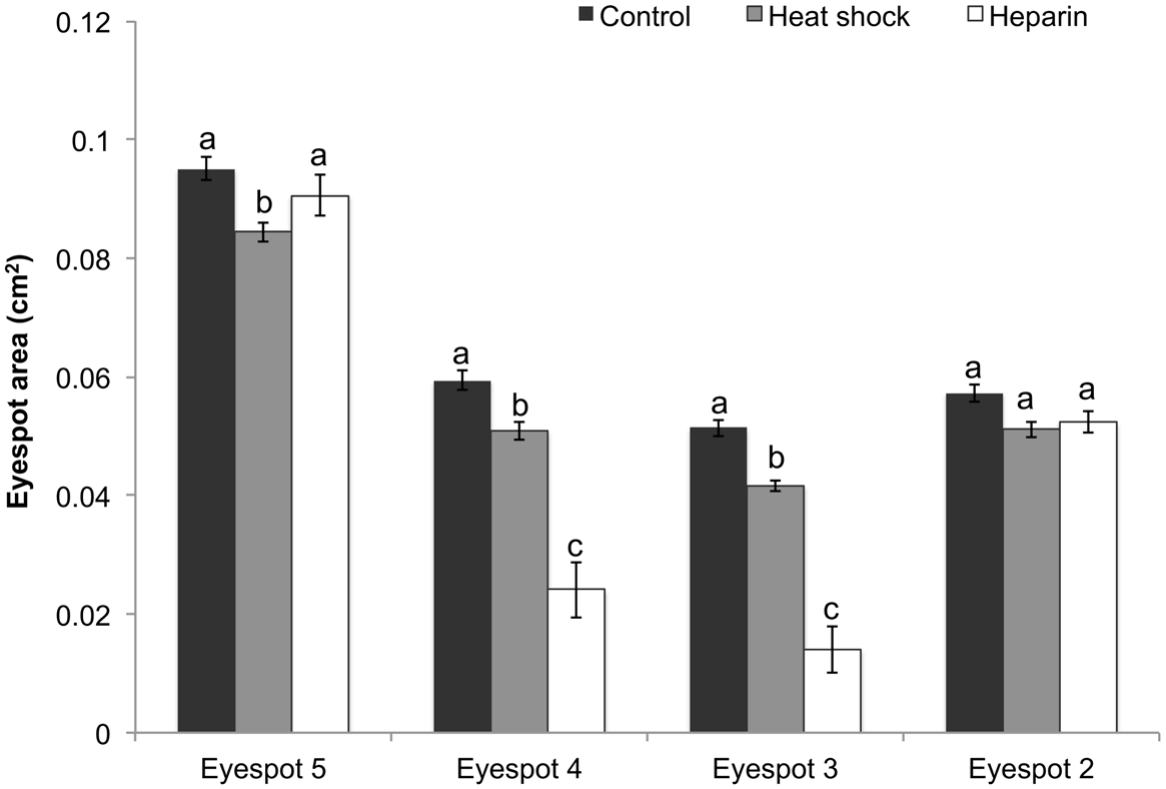
Eyespot size across all four eyespots in the different treatments. Data represent the area in cm^2^ with error bars representing 1 SE from the mean. The 2-way ANCOVA was performed on square-root transformed data. Results shown in Table 1. Different letters above the bars indicate significant differences within each individual eyespot.

For eyespot area, there was no batch by treatment interaction (F _(2, 94)_ = 1.6, p = 0.2) or batch by wing interaction (F _(2, 380)_ = 1.73, p = 0.17); however, there was a treatment by eyespot interaction (F _(2, 380)_ = 27.2, p < 0.0001). Within the control group, eyespot 5 was significantly larger than eyespots 2, 3 and 4. Eyespot 4 was larger than eyespot 3 although there was no difference in size between eyespots 3 and 4 compared to eyespot 2. The treatments had variable effects on eyespot size; heparin dramatically reduced the size of eyespots 3 and 4 often eliminating them entirely while it had no effect on the size of eyespots 2 and 5 (Fig 3). Heat shock significantly reduced the size of all eyespots with the exception of eyespot 2.

### Black Border (Outer Ring)

**Fig 4:**
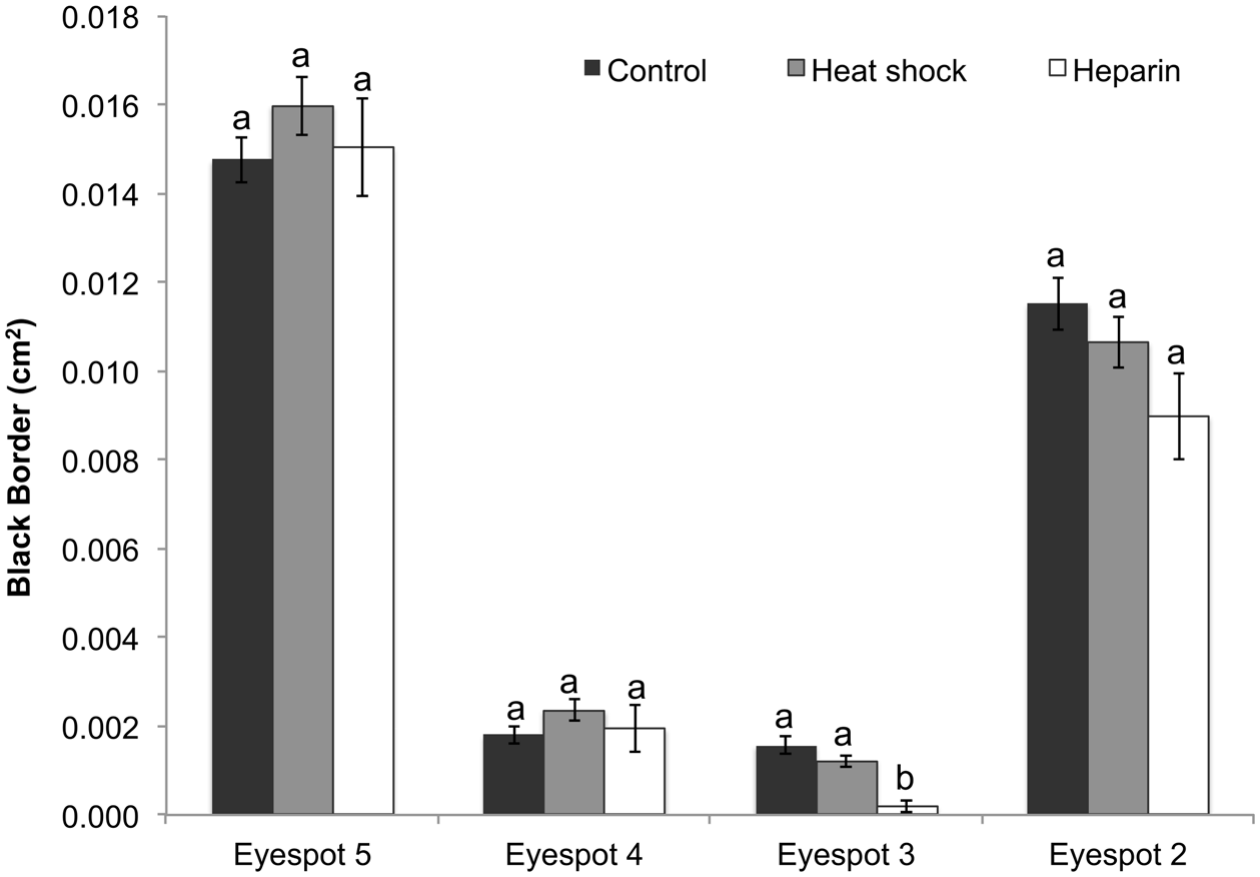
Area of the black border (outer ring) across all four eyespots in the different treatments. Data represent the area in cm^2^ with error bars representing 1 SE from the mean. The 2-way ANCOVA was performed on square-root transformed data. Results shown in Table 1. Different letters above the bars indicate significant differences within each individual eyespot.

There was no batch by treatment interaction for the replicated experiments (F _(2, 348)_ = 2.0, p = 0.12). Within the control group, the area of the black border varies across all eyespots. Eyespot 5 exhibits the largest border, followed by eyespot 2 with the two central eyespots possessing the smallest black border. A significant treatment by eyespot interaction was found (F _(6, 424)_ = 4.62, p<0.0001). However, neither treatment had an effect on the area of this eyespot ring for most eyespots overall, with the exception of butterflies treated with heparin. These butterflies had a significantly smaller black border in eyespot 3 compared to the control group (Fig 4).

### Yellow Pigment

**Fig 5:**
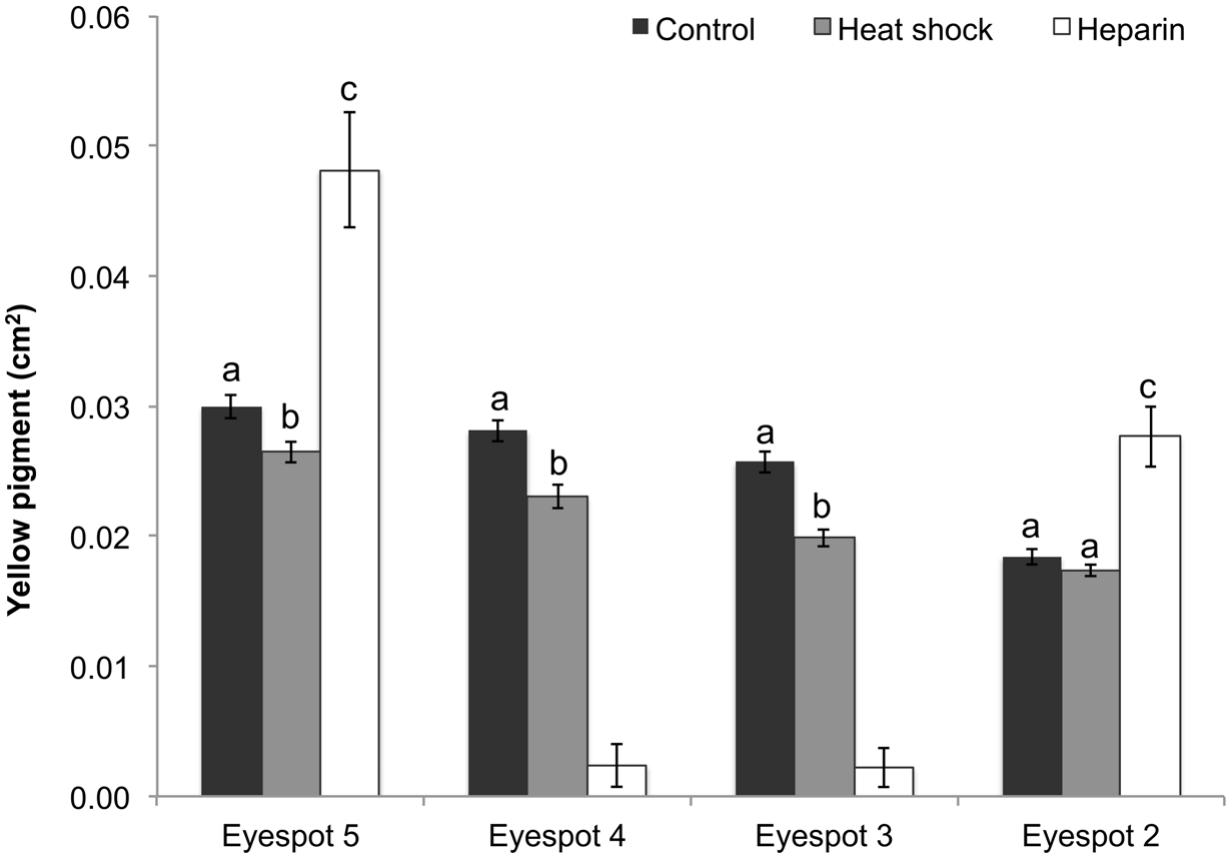
Area of yellow pigment across all four eyespots in the different treatments. Data represent the area in cm^2^ with error bars representing 1 SE from the mean. The 2-way ANCOVA was performed on log10-transformed data. Eyespots 3 and 4 in the heparin treatment were excluded from the statistical analysis, as eyespots were mostly abolished by the treatment. Results shown in Table 1. Different letters above the bars indicate significant differences within each individual eyespot.

Due to the strong effects of heparin on eyespot pigmentation, the analysis of yellow pigment was conducted first by examining the effects of heat shock on all eyespots, and then by examining the effect of both treatments on eyespots 2 and 5. No batch by treatment interaction was found for the replicated experiments (F _(2, 348)_ = 0.99, p = 0.3). There was a significant treatment by eyespot interaction (F _(6, 348)_ = 5.15, p = 0.0017). In the control group, the amount of yellow pigmentation exhibited a gradual decline in area over the four eyespots, with the largest amount of yellow pigment observed in eyespot 5 and the smallest amount in eyespot 2. Heat shock had a marginally significant effect on reducing the area of yellow pigmentation in eyespot 5 (Fig 5). There was a significant reduction in the area of yellow pigment in eyespots 3 and 4. Heat shock had no effect on the area of yellow pigment in eyespot 2.

A separate analysis including heparin treatment was done for eyespots 2 and 5 as heparin abolished eyespots 3 and 4 in many butterflies. For this comparison, there was no treatment by eyespot interaction for yellow pigmentation in eyespots 2 and 5 likely due to high variability among individuals (F _(2, 212)_ = 0.95, p = 0.3885).

### Orange Pigment

**Fig 6:**
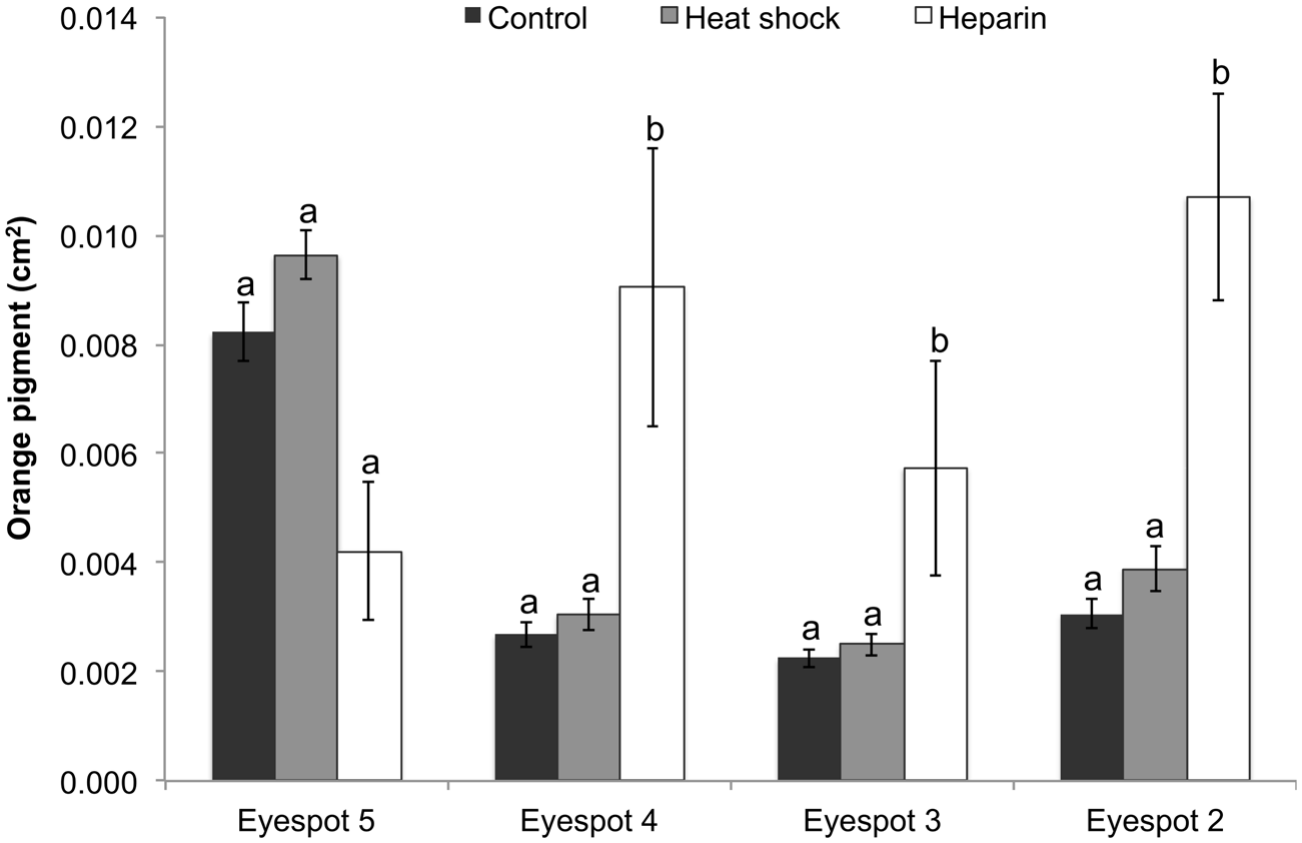
Area of orange pigment across all four eyespots in the different treatments. Data represent the area in cm^2^ with error bars representing 1 SE from the mean. The 2-way ANCOVA was performed on log10-transformed data. Results shown in Table 1. Different letters above the bars indicate significant differences within each individual eyespot.

The analysis revealed no batch by treatment interaction (F _(2, 344)_ = 1.6, p = 0.2). However, there was a significant treatment by eyespot interaction (F _(6, 387)_ = 10.8 p < 0.0001). Within the control group, the area of orange pigmentation was significantly larger in eyespot 5 compared to eyespots 2, 3 and 4, while no difference was observed among eyespots 2 and 3. Heat shock had no detectable effect on the area of orange pigment in any eyespot (Fig 6) Heparin significantly reduced the area of orange pigment in eyespot 5, but increased this color in eyespots 2, 3 and 4 (Fig 6 and S1 Fig).

### Blue Scales

**Fig 7:**
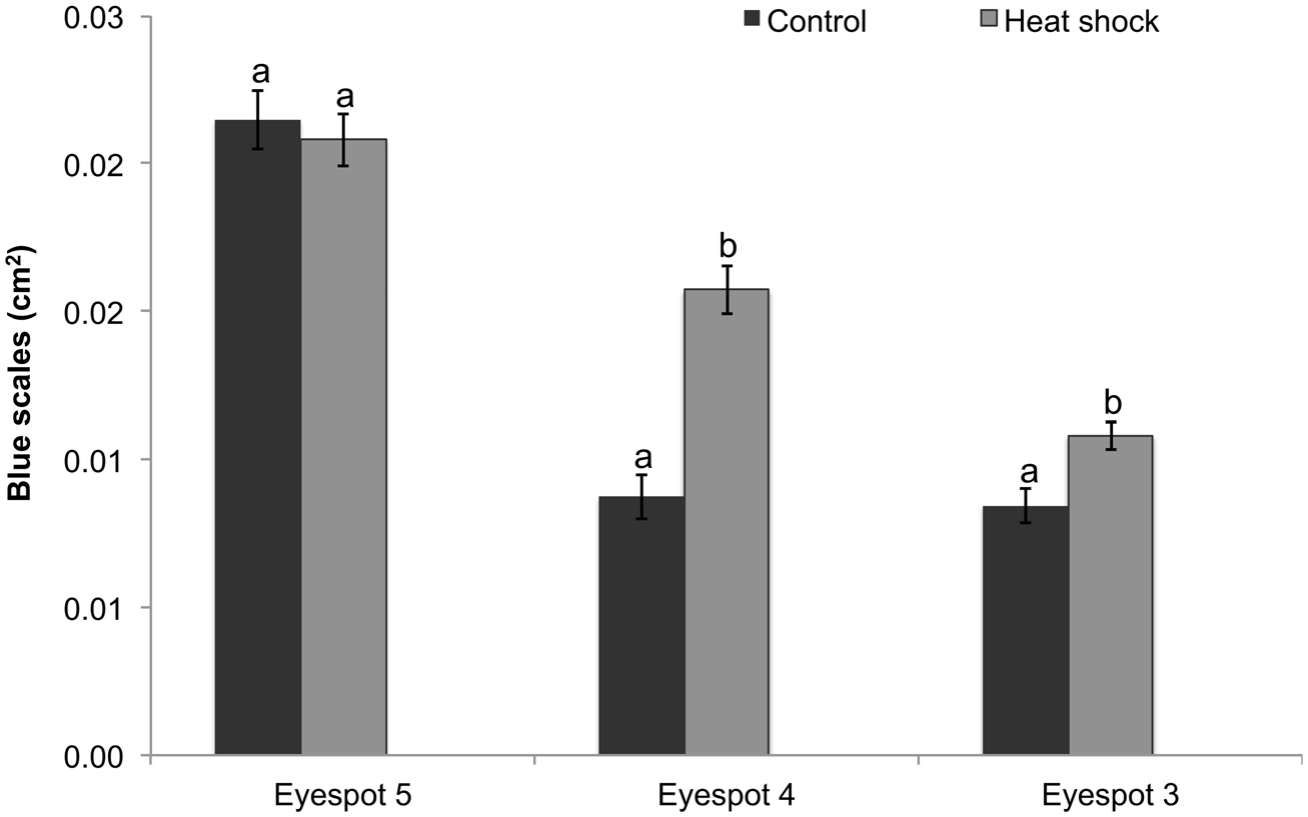
Area of blue scales across all four eyespots in the different treatments. Data represent the area in cm^2^ with error bars representing 1 SE from the mean. The 2-way ANCOVA was performed on square-root transformed data. Results shown in Table 1. Different letters above the bars indicate significant differences within each individual eyespot.

Heparin virtually eliminated blue scales in all eyespots (Fig 1); thus, only the heat shock and control groups were compared for eyespots 3–5 as this structural color is rarely observed in eyespot 2. There was no batch by treatment interaction (F _(2, 261)_ = 0.9, p = 0.4); however, there was a significant treatment by eyespot interaction (F _(2, 264)_ = 15.08, p<0.0001). Within the control group, the area of blue scales was significantly higher in eyespot 5 versus eyespots 3 and 4. No difference observed between eyespots 3 and 4. Heat shock significantly increased the area of blue scales in eyespots 3 and 4 but had no effect on eyespot 5 (Fig 7).

### Black Focus

**Fig 8:**
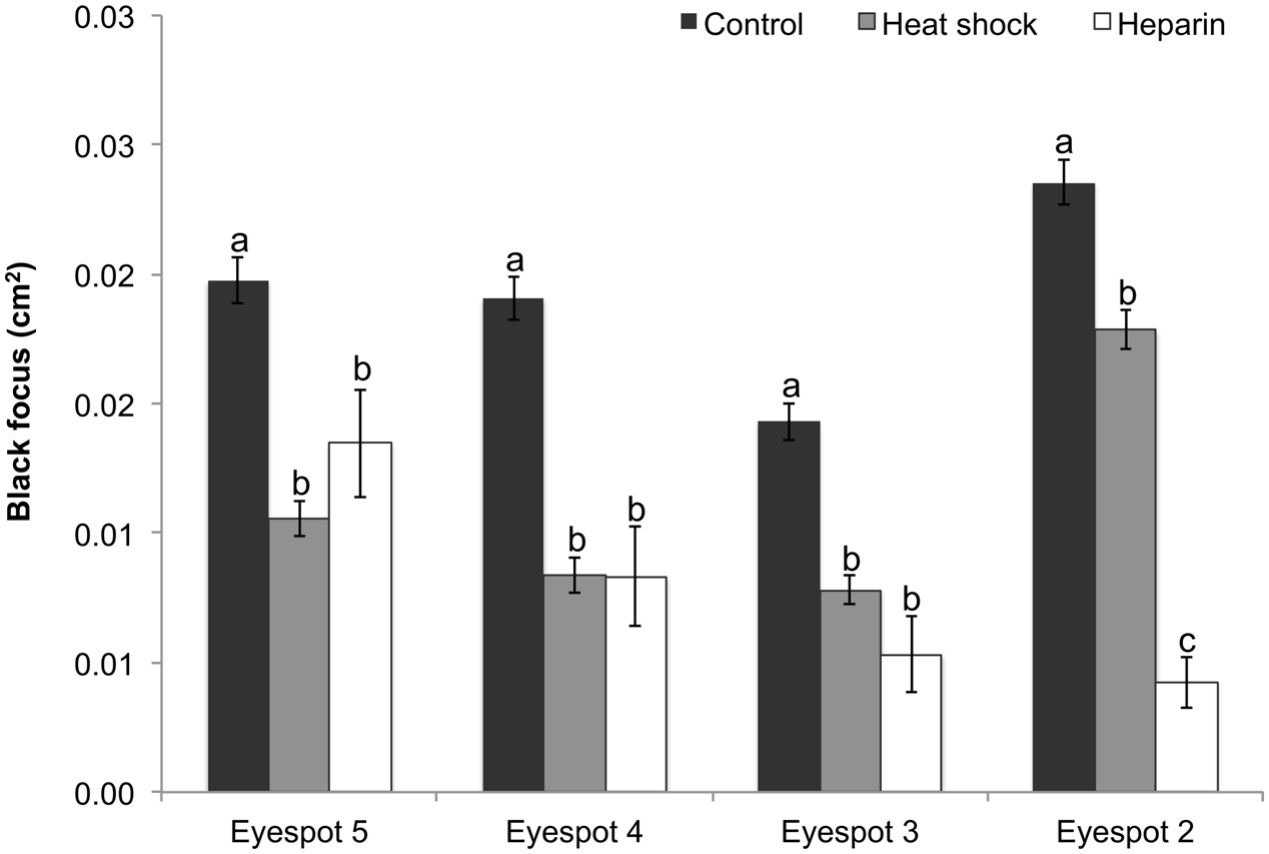
Area of the black focus across all four eyespots in the different treatments. Data represent the area in cm^2^ with error bars representing 1 SE from the mean. The 2-way ANCOVA was performed on square-root transformed data. Results shown in Table 1. Different letters above the bars indicate significant differences within each individual eyespot.

For the black focus, there was no batch by treatment interaction (F _(2, 349)_ = 0.7, p = 0.5). There was a significant treatment by eyespot interaction (F _(2, 425)_ = 13.77, p < 0.0001). In the control butterflies, the area of the black focus varied significantly across eyespots. There was no difference in the area of the black focus between eyespot 5 and 4; however, comparisons between all other eyespots were significantly different with the smallest focus observed in eyespot 3 and the largest in eyespot 2. Heparin significantly reduced the size of the black focus in all eyespots with a dramatic reduction in eyespot 2. Heat shock significantly reduced the size of the black focus in all eyespots with the strongest effect observed in eyespot 4 (Fig 8).

### Tests for Modularity and Integration

**Fig 9:**
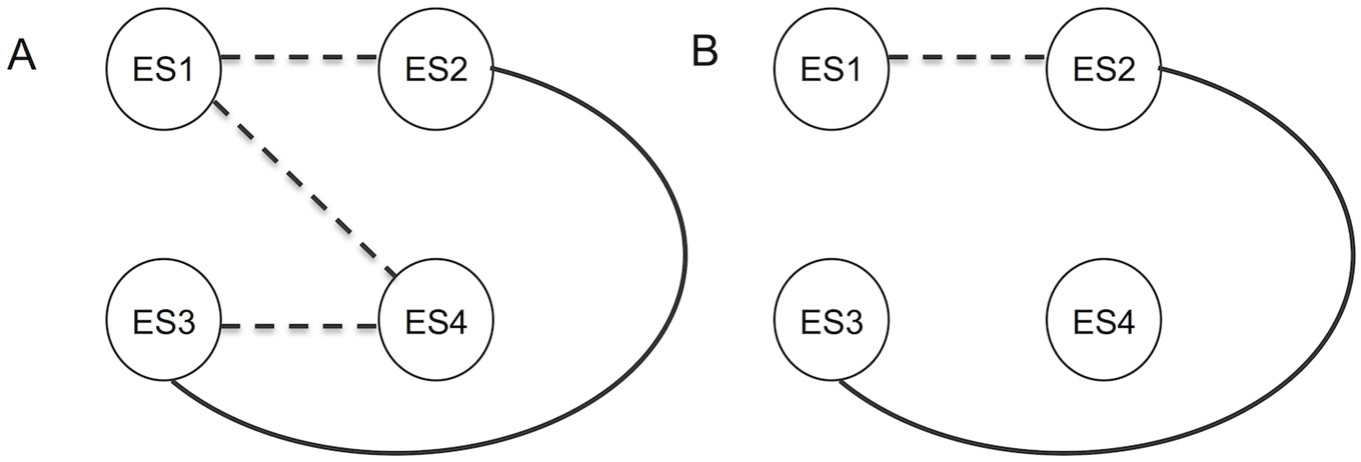
Hypotheses of integration and modularity for control eyespots based on overall phenotype data. **A** represents the most intergrated model inferring weak integration between neighboring eyespots and between eyespots 2 and 5 (for the black border). **B** represents the most conservative model showing weak integration only between eyespots 4 and 5. Broken line represents weak integration and the solid line represent strong integration.

**Fig 10:**
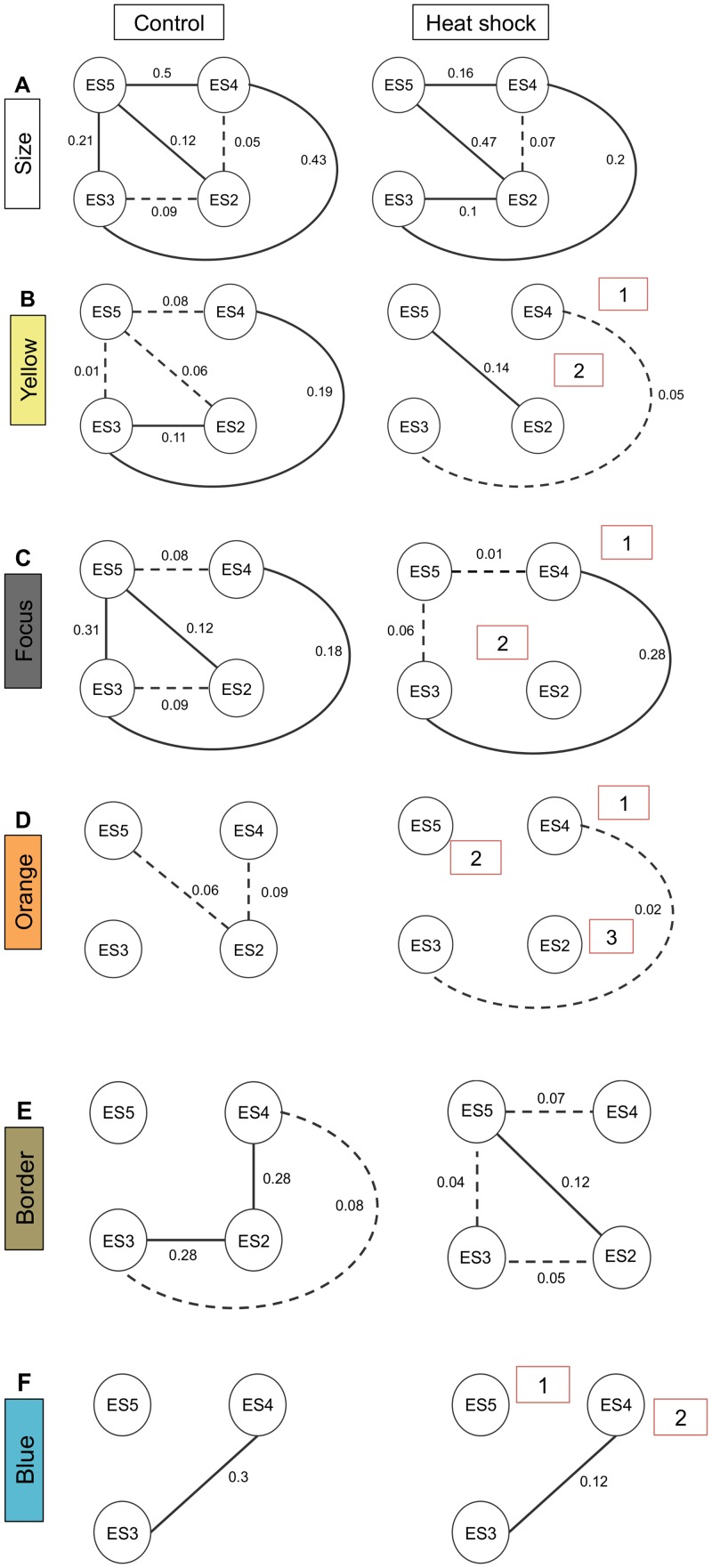
Comparison of phenotypic correlations within and between control and heat shock groups for eyespot size and pigment rings. Edges infer integration among eyespots based on edge exclusion deviance values above the critical value from the χ^2^ distribution. Eyespots without edges are conditionally independent from all other eyespots, χ^2^ <3.84, p<0.05. Edge strength values represent the strength of integration between eyespots [32]. Heat shock results in a doubling of independent modules (shown by red numbered boxes). Solid and broken lines represent strong and weak integration respectively.

Based on results from the phenotype data, we tested the prediction that eyespots 3 and 4 form an independent module that exhibits weak integration between adjacent and morphologically similar eyespots 4 and 5. Eyespot 2, which is morphologically differentiated, would represent a further independent module that also may exhibit weak integration with eyespot 5 for the black border (Fig 9). Matrices for partial correlations and EED values are presented in S3 and S4 Figs respectively. The graphical model for control eyespots reveals that eyespot size in *V*. *cardui* is highly integrated across eyespots (Fig 10A). The strength of integration is highly variable with eyespots 2 and 4 exhibiting the weakest edge strength. Overall, the yellow pigment (Fig 10B) and black focus (Fig 10C) display similar integration patterns across eyespots with a lack of integration between eyespots 2 and 4. Only orange pigment (Fig 10D) and the black border (Fig 10E) showed evidence of integration between these two eyespots, and overall exhibited the lowest levels of integration.

These results suggest that the eyespots are largely integrated for many traits, with some eyespots demonstrating modularity for certain pigments. Following heat shock, patterns of integration were largely intact for eyespot size, with only one edge removed between eyespots 3 and 5 (Fig 10A). Edge strengths weakened for some eyespots but increased for others. The colored eyespot elements displayed very different patterns of integration compared to controls with an overall loss of integration among eyespots resulting in a doubling of independent modules. The yellow pigment (Fig 10B) and black focus (Fig 10C) were reduced to two independent modules and the orange pigment (Fig 10D) was reduced to three. Examination of the blue element revealed an independent module composed of eyespots 3 and 4 in both the control and heat shock group (Fig 10F). These modules reflect changes in the proportions of color for heat shock relative to the control (Table 2).

**Table 2:** Percent difference in area (cm^2^) for eyespot size and color in response to heat shock (37°C, 48 hrs.) relative to the control. Module number represents groups identified using graphical modeling (Fig 10) that corresponds to percent changes in size and color.

	Eyespot no.	Heat shock	Module
**Eyespot size**	5	-11.2	0
	4	-14.7	0
	3	-19.0	0
	2	-10.9	0
**Black focus**	5	-46.5	1
	4	-56.1	1
	3	-45.5	1
	2	-24.2	2
**Blue**	5	-3.2	1
	4	44.3	2
	3	21.8	2
**Orange**	5	14.6	1
	4	11.7	2
	3	10.0	2
	2	21.1	3
**Yellow**	5	-11.7	1
	4	-18.1	2
	3	-22.8	2
	2	-5.6	1
**Black border**	5	7.6	0
	4	23.3	0
	3	-22.4	0
	2	-9.4	0

The black border exhibited an increase in patterns of integration between eyespots. A new edge was created between eyespots 3 and 4 for the orange pigment. Overall, in both the control and treatment groups, the two central eyespots retained the highest number of edges between each other across all traits (Table 3) and displayed some of the highest edge strength values. We also observed a general trend of concerted changes in response to the treatment. Yellow pigment and the black focus declined in response to heat shock across all eyespots while the orange pigment and area of blue scales increased.

**Table 3:** Total number of edges across all traits between each pair of eyespots for the control and heat shock (37°C, 48 hrs.) groups. The two central eyespots (ES3 and ES4) are highlighted as showing the highest number of edges and no change in edge number following heat shock.

	Control	Heat shock	Difference
ES5 vs. ES4	3	3	0
ES5 vs. ES3	3	2	-1
ES5 vs. ES2	4	3	-1
**ES3 vs. ES4**	**5**	**5**	**0**
ES2 vs. ES4	4	2	-2
ES2 vs. ES3	3	1	-1

## Discussion

Our morphometric analysis revealed that *V*. *cardui* ventral hindwing eyespots are generally integrated for eyespot size and several color elements across the wing. However, exposure to novel conditions can disrupt these patterns of integration. In *V*. *cardui*, heat shock has subtle but significant effects on eyespot development, while heparin dramatically altered eyespot morphology. Despite using these two very different types of perturbation, we observed common effects on eyespots 3 + 4, indicating that some underlying property of the central eyespots influences pattern modification. The perturbation experiments also identified other eyespots and features that exhibited patterns of modularity. Graphical modeling of heat shock experiments also suggest that 1) phenotypic correlations are not static and can change when organisms are exposed to novel conditions and 2) the response to perturbation can reveal cryptic variation in levels of integration which may reflect differences in the expression of regulatory molecules across the wing.

### Heat Shock and Heparin Reduce Eyespot Size

Our experiments revealed that both treatments had significant and variable effects on the size of different eyespots independent of wing area. Heat shock significantly reduced the size of eyespots 3–5 while heparin significantly reduced the size of eyespots 3 and 4. While similar reductions in eyespot size have been demonstrated following heparin injections in another nymphalid, *Junonia coenia*, [39] temperature shifts have opposite effects in other butterfly species including *B*. *anynana* [37,49,50] and *J*. *alamana* [51]. Thus, temperature seems to have complex effects on butterfly eyespot development, increasing eyespot size in some species while decreasing it in others. Neither heat shock or heparin significantly affected the size of eyespot 2, suggesting aspects of its development are under modular control and more canalized than those of the other eyespots. These results support previous observations that sensitivity of pattern elements to experimental perturbations appears to be influenced by their position on the wing [28].

Grafting experiments have revealed that eyespot size is regulated by some property of the focal organizing cells such as a morphogen signal [11,17,52]. Thus, warmer temperatures may increase the concentration of this signal in *B*. *anynana* and *J*. *almana* while weakening the focal signal in *V*. *cardui*. Research in *B*. *anynana* has shown that while changes in temperature do not alter the temporal order of gene expression for eyespot associated genes, cooler temperatures lead to earlier onset of gene expression [49]. Thus, temperature may induce heterochronic shifts in expression of eyespot-associated genes with potential effects on eyespot size [49]. In addition to temperature-induced variation in expression of eyespot genes, hormone titers have also been implicated in regulating eyespot size plasticity [53]. In a recent study of *B*. *anynana*, the area of several eyespot rings were significantly increased following pupal injections of the hormone 20-hydroxyecdysone [37]. Therefore heat shock and heparin may have reduced eyespot size in *V*. *cardui* by altering the dynamics of hormone signaling and/or eyespot genes, or by modifying the competency of scale cells to respond appropriately to morphogen signals [26]. Whatever the mechanism, eyespots 3 + 4 were most strongly affected in both treatments.

### Inner Eyespot Elements Are More Sensitive to Perturbations Than the Eyespot Border

The heparin treatment virtually eliminated eyespots 3 + 4 and strongly impacted the inner pattern elements. In many butterflies, heparin produced a bleaching effect or distortion of the inner eyespot elements, merging them distally with parafocal elements (Fig 1 and S5 Fig). In contrast, the black eyespot border, particularly the proximal region is somewhat resistant to this perturbation. Similar effects of heparin have also been reported in eyespots of *Junonia coenia* [39]. Heparin does not induce similar modifications in all butterfly species [45,46]. In *Heliconius* butterflies, heparin results in dose-dependent expansion of black pigmentation across the wing [46]. Heparin-induced modifications correspond to *WntA* expression, suggesting heparin regulates melanin pigmentation in *Heliconius* by promoting diffusion of this signaling molecule [46]. In *V*. *cardui*, heparin expands black pigmentation of the parafocal elements on the dorsal surface of the hindwings; but bleaches the dorsal black spots (S6 Fig). The effects of heparin, therefore, depend on the position of pattern elements on the wing, with increased sensitivity in the distal region and eyespot centers. Heparan sulfates are known to bind morphogens and influence their diffusion [54,55]. Thus, heparin effects on eyespot development may be due to disruption in morphogen signaling, although grafting experiments suggest that heparin can alter wing patterns independently of morphogen signaling and may instead affect some other regulatory molecule that influences pigmentation [39]. It has been proposed that this regulatory molecule may be a cold shock hormone produced outside of the wing that can alter threshold levels of morphogens without changing their positional information [39,56]. Support for a putative cold shock hormone has been shown in transfusion experiments where a mild cold shock phenotype was induced in butterflies receiving hemolymph from cold shock pupa [44].

It is intriguing that the heparin phenotype bears a striking resemblance to the effects of cold shock [40], of sodium tungstate in *V*. *cardui* [29], and even occasional aberrant forms observed in nature. Moreover, these phenotypes all demonstrate significant modification of inner eyespot elements and relative canalization of the outer border. Concordance among phenotypes induced by these entirely different perturbations strongly suggests evidence of developmental modularity between the inner and outer regions of the eyespot field.

Heat shock also altered patterning of the inner eyespot color elements. The black focus was significantly reduced in all eyespots, with the strongest effects in the two central eyespots. In contrast, the area of the blue element was significantly increased in the two central eyespots, particularly for eyespot 4. In the two central eyespots the black focus appears to have been partially replaced or masked by expansion of the metallic blue scales. The blue region may have expanded at the expense of the yellow ring, which also decreased in response to heat shock. In *J*. *orithya*, heat shock has the opposite effect and reduces the area of blue scales on the wing, confirming previous observations that heat shock induced modifications appear to be species-specific [57].

Replacement and expansion of pigment rings has also been observed in other butterflies. Genetic studies have identified changes in expression of patterning genes following expansion of pigment rings. In the Goldeneye mutant of *B*. *anynana*, the inner ring of black scales was replaced by expansion of gold scales, which corresponds to changes in the expression domains of transcription factors. In these mutants Spalt expression, which maps to black scales was replaced by expression of Engrailed/Invected, which map to gold scales [13]. A similar mechanism may explain expansion of blue scales in *V*. *cardui* eyespots following heat shock. Spalt and Engrailed/Invected expression maps to black pigmentation in *V*. *cardui* [13]. Thus, heat shock may repress expression of these transcription factors resulting in development of blue scales. Immunolabeling experiments would be required to test this hypothesis.

### Heat Shock Disrupts Patterns of Integration Revealing Modularity in Eyespot Development

We predicted that patterns of integration and modularity present in control butterflies would explain eyespot responses to perturbation. If eyespots or color elements are integrated, these may show coordinated responses to heat shock, in contrast to eyespots/color elements exhibiting modularity. We assumed that eyespots 3 + 4 formed an independent module and had either no or weak integration with eyespots 2 + 5. The graphical models revealed that most traits were largely integrated across all eyespots with some evidence of modularity in eyespots 3 + 5 in control butterflies. Therefore, these models did not predict the modular responses to perturbation: the two central eyespots were integrated with the other eyespots in the control butterflies, a pattern that is also observed for ventral hindwing eyespots in other butterflies [33,58]. Following heat shock, the two central eyespots exhibited strong modularity retaining their connections to each other for most traits; in contrast to the other eyespots where the different color elements became largely uncoupled forming separate modules. Therefore, the strength of integration varies across the wing, with strong coupling between eyespots 3 + 4, indicating a certain degree of modularity, revealed only when pupa are exposed to novel conditions.

Previous studies have documented strong correlations for eyespot size between adjacent eyespots [33,58]. Co-variation for some color elements and eyespot size was also observed between neighboring eyespots in *V*. *cardui*. Following heat shock, patterns of integration were maintained for size but not for the color elements. Thus, heat shock alters phenotypic correlations among traits and produces new correlation patterns that were not present in the controls. These findings support other studies that demonstrate correlations among traits can change when organisms are exposed to novel conditions [59–62]. Such changes in phenotypic correlations could facilitate pattern diversification if novel conditions promote modularity and un-coupling of pattern elements during development. Whether changes in phenotypic correlations influence pattern diversification in Lepidoptera remains unknown although expansion into novel environments has been proposed as a contributing factor driving wing pattern evolution in the genus *Vanessa* [29,43,56,63].

### Do Gradient Models Adequately Explain Eyespot Development?

Results reported here and in other studies [17,44] indicate that the outer ring of eyespots is less vulnerable to perturbations than the inner pigment rings. These observations suggest that expression of morphogens or transcription factors are less stable in the focal region. For *V*. *cardui*, the most sensitive eyespot element appears to be the black focus, which was significantly modified in all eyespots by both treatments. Several studies have shown that heat shock can reduce melanin pigmentation [43,64]. In the case of *V*. *cardui* eyespots, it is not melanin *per se* that is affected, but the location of this pigment that influences its susceptibility to perturbation. These results raise two important questions regarding the development of eyespot elements. First—how can a concentration gradient produce the same color in both the focus and the outermost ring? Second—how do perturbations significantly modify the inner eyespot elements without affecting the outermost ring?

According to classical gradient models which are based on ideas developed by Wolpert [65], the eyespot develops from an organizing focus, which contains the highest concentration of a putative morphogen signal. This morphogen diffuses radially through the wing epidermis to produce concentric rings of colored scales [17,27]. Thus, each ring represents a different threshold response to the diffusing morphogen, resulting in a signal transduction cascade leading to the development of different colored rings or pattern elements [19].

A simple concentration gradient model does not appear to explain eyespot patterns in *V*. *cardui*. According to this model both low and high concentrations of the putative morphogen leads to the same colored pigment, suggesting that the focus and outer border are producing melanin via alternative mechanisms. Spatial differences in interactions between hormones and transcription factors could influence how epidermal cells interpret the signal, leading to synthesis of the same pigment. Fluorescent labeling of eyespot 2 in 24 hr pupae of *V*. *cardui* shows that different genes are expressed in the inner and outer rings with co-expression of Distal-less and Spalt in the focus and Engrailed/Invected in the outer ring [13]. These different expression profiles could explain why the outer ring is less vulnerable to perturbation if expression of certain transcription factors is developmentally buffered. It also suggests that different patterning genes are involved in producing the same pigment, in this case melanin. However, expression patterns vary substantially during wing color pattern development [66] and we do not yet have a detailed time series of expression patterns during eyespot development.

Variation in threshold responses to a concentration gradient cannot easily explain the same pigment developing in different rings. Neither can it account for the development of distorted or asymmetric eyespots observed in some butterfly species, which may be better explained by a reaction-diffusion model [15,51]. The sensitivity of the inner pattern elements relative to the outer border is also difficult to reconcile with a simple model of a single diffusing morphogen. It is possible that both the concentration gradient and reaction-diffusion models explain eyespot development, either independently in different species or even simultaneously in the same species. Recent proposals suggest that these two models should not be considered to operate exclusively and may in fact collaborate to generate morphological complexity and diversity [67]. Perhaps coordination of these two patterning mechanisms across different morphogenic fields promotes flexibility and modularity during development. Further work is required to understand how such patterning models can adequately explain the enormous diversity of butterfly eyespots.

### Developmental Coupling Between the Two Central Eyespots

One of the main findings from this study is the apparent developmental coupling of eyespots 3 + 4. Coupling of these two eyespots is also observed in phylogenetic studies and mutation experiments involving multiple butterfly species. Phylogenetic analyses examining eyespot evolution in both *Vanessa* and *Junonia* reveal that although the ancestral state was likely a serial eyespot arrangement, evolutionary patterns of eyespot loss in different lineages always involved eyespots 3 + 4 [47,48]. Two mutants were also discovered in *B*. *anynana* where eyespots 3 + 4 are either reduced or absent with no effect on other eyespots or wing patterns [22,68]. One of these mutants, *Missing* also showed highly reduced expression of Distal-less and Engrailed [68]. Taken together these studies strongly suggest that coupling of eyespots 3 + 4 is a general phenomenon across nymphalids, involving both genetic and developmental mechanisms such as shared cis-regulatory elements controlling focus differentiation and regional expression of regulatory molecules across these two wing sectors [22]. These two mechanisms could explain the trends observed across phylogenetic studies, mutation experiments and physiological perturbations. Future work on cis-regulatory control of eyespot development may elucidate how eyespot development is regulated and why certain eyespots are more sensitive to modification or loss in both developmental and evolutionary contexts.

## Supporting Information

S1 Fig Heparin-induced orange pigmentation. Heparin increased orange pigmentation in the eyespots of many individuals. This effect was observed in all eyespots with the exception of eyespot 5.
(TIFF)

S2 Fig Bivariate analysis of the sum of all pigments in each eyespot relative to the total eyespot area. Plots reveal a strong correlation in the measurements of the pigment area to the total eyespot area.
(TIFF)

S3 Fig Partial correlation matrices for eyespot size and proportion of each eyespot pattern element. Values below the diagonal represent control eyespots and those above represent the heat shock treatment. Wing area was used as a covariate. Partial correlations are used to measure the association (or integration) between pairs of traits, independent of associations with all other measured traits (Magwene, 2001, Allen 2008).
(TIFF)

S4 Fig Edge exclusion deviance matrices based on partial correlations and calculated using the EED formula described in the methods. EED uses partial correlations to test for conditional independence. Values highlighted in bold (<3.82) suggest conditional independence and values >3.82 indicate eyespots are integrated. Values below the diagonal represent control eyespots and those above represent the heat shock treatment.
(TIFF)

S5 Fig Range of phenotypes induced by Heparin injection. Butterflies exhibit a range of responses to heparin including complete loss of eyespots as shown in Fig 1, to bleaching and distortion of inner pattern elements with parafocal elements. A similar range of phenotypes is also observed following exposure to cold shock and sodium tungstate [29,40].
(TIFF)

S6 Fig Effect of heparin on hindwing dorsal patterns. Heparin expands black pigmentation of parafocal elements and eliminates black pigment in dorsal spots.
(TIFF)
