# Supplementary figures and images for: Physiological Perturbation Reveals Modularity of Eyespot Development in the Painted Lady Butterfly, Vanessa cardui
Source: PLoS One. 2016 Aug 25;11(8):e0161745. doi: 10.1371/journal.pone.0161745 (PMC4999082; doi:10.1371/journal.pone.0161745)

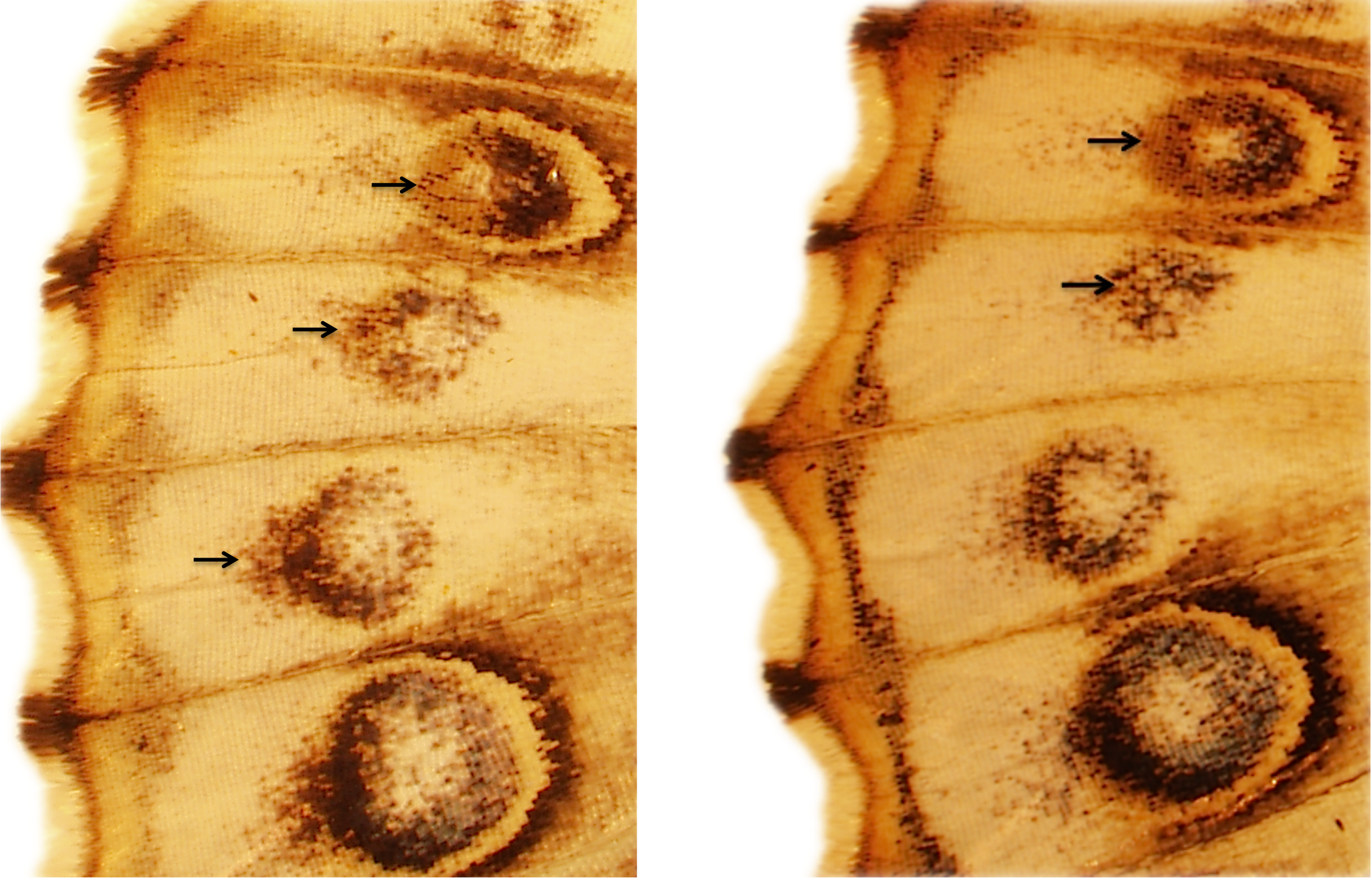

Supplement: S1 Fig — Heparin increased orange pigmentation in the eyespots of many individuals. This effect was observed in all eyespots with the exception of eyespot 5. (TIFF) [file pone.0161745.s001.tiff]

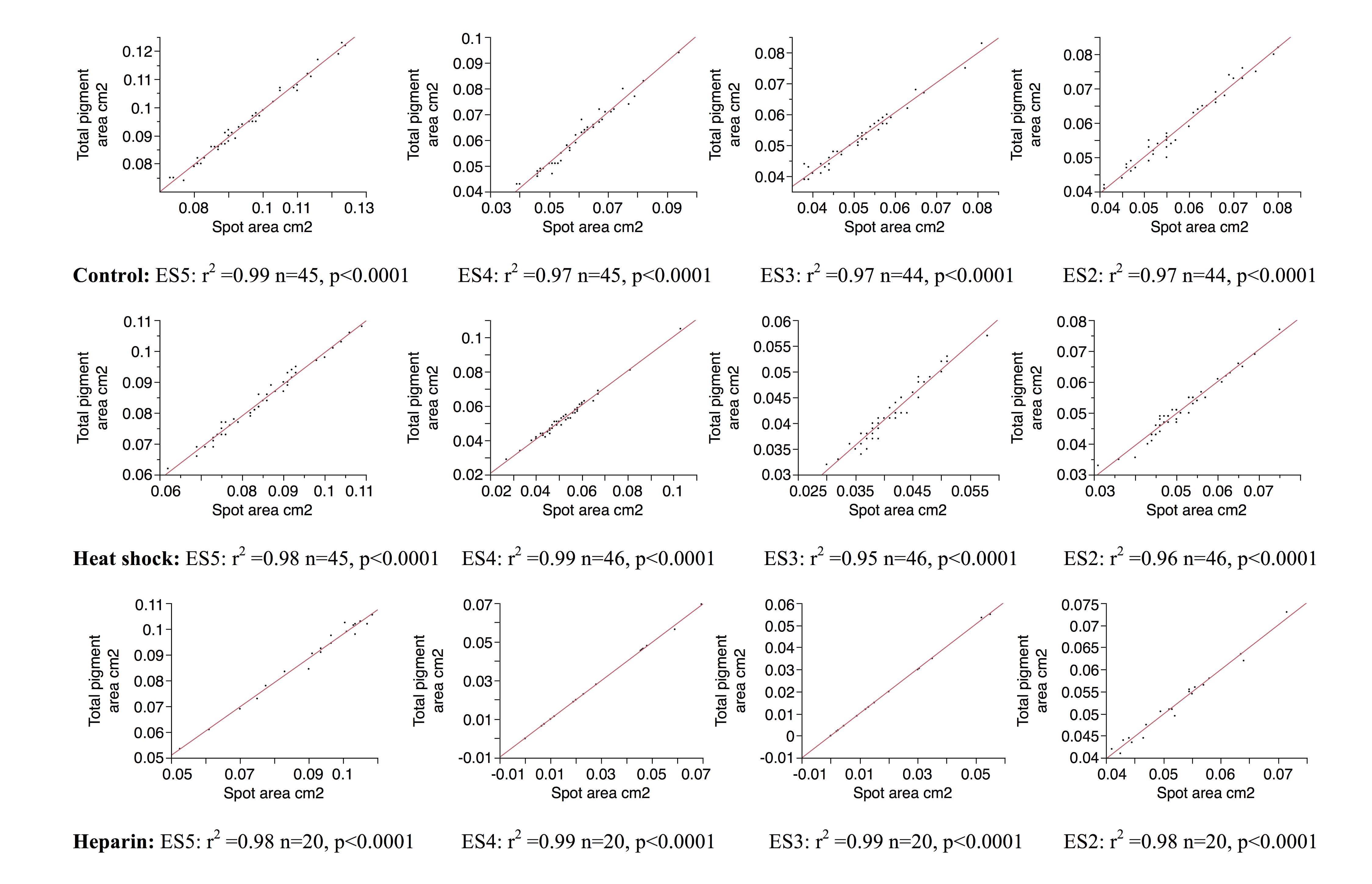

Supplement: S2 Fig — Plots reveal a strong correlation in the measurements of the pigment area to the total eyespot area. (TIFF) [file pone.0161745.s002.tiff]

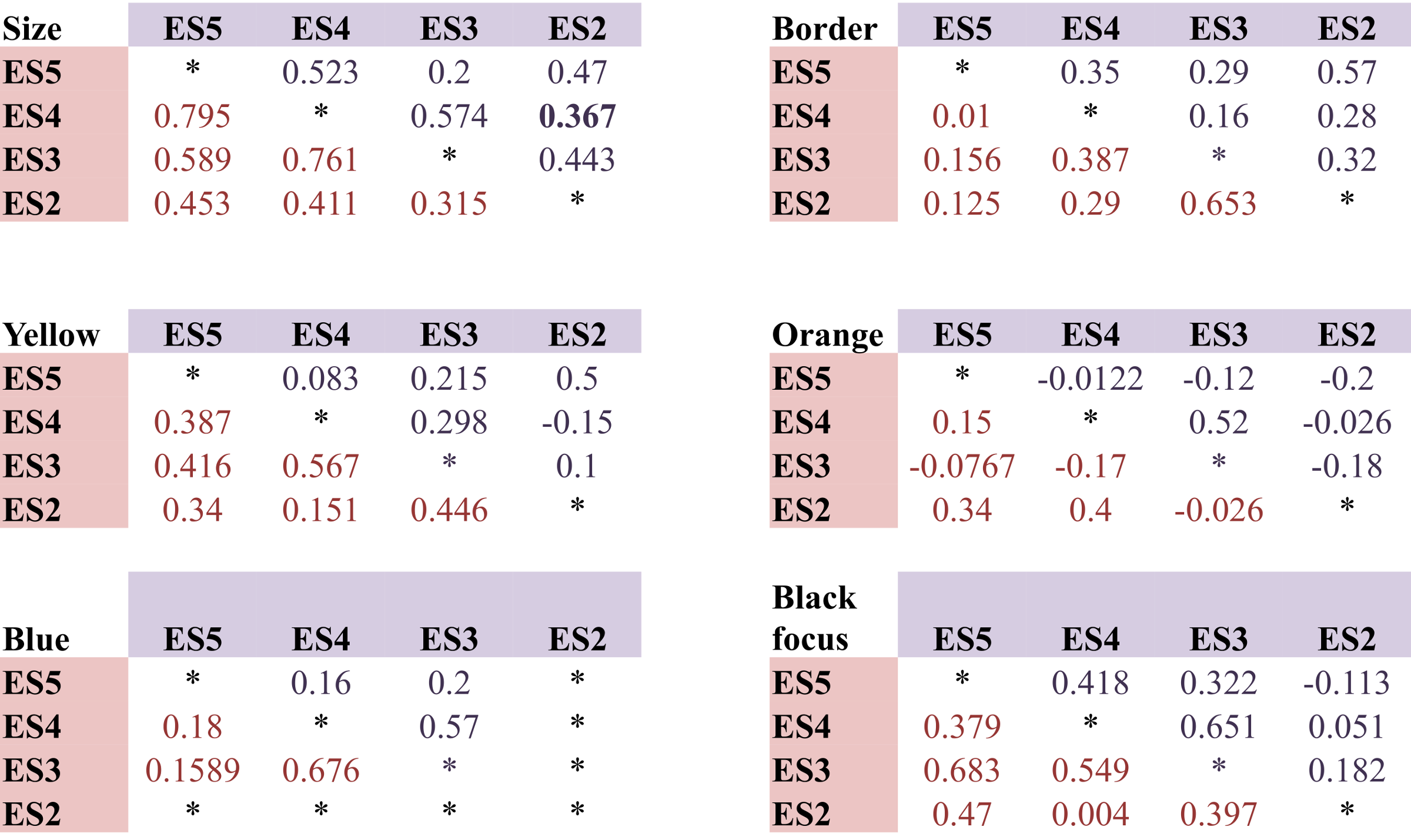

Supplement: S3 Fig — Values below the diagonal represent control eyespots and those above represent the heat shock treatment. Wing area was used as a covariate. Partial correlations are used to measure the association (or integration) between pairs of traits, independent of associations with all other measured traits (Magwene, 2001, Allen 2008). (TIFF) [file pone.0161745.s003.tiff]

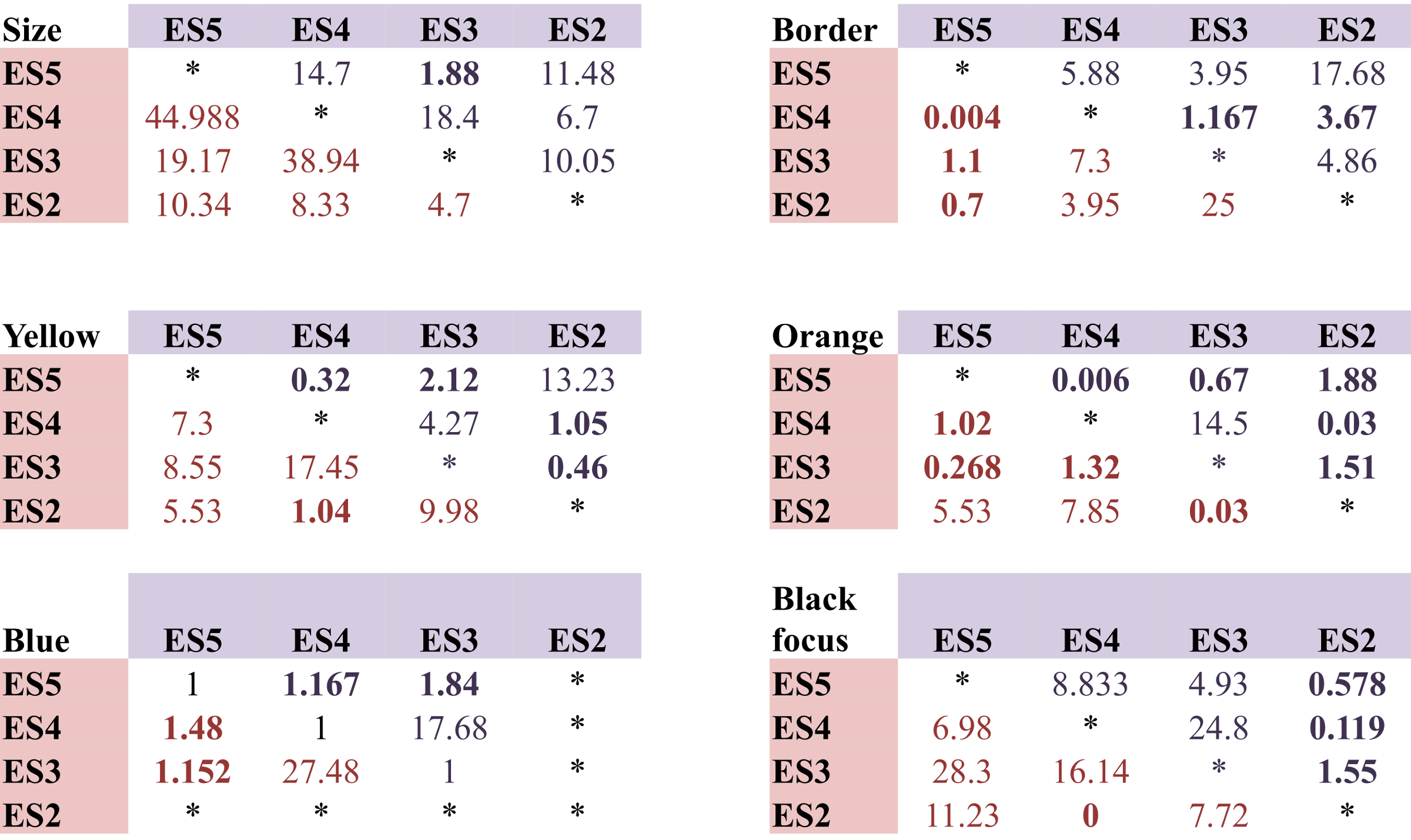

Supplement: S4 Fig — EED uses partial correlations to test for conditional independence. Values highlighted in bold (<3.82) suggest conditional independence and values >3.82 indicate eyespots are integrated. Values below the diagonal represent control eyespots and those above represent the heat shock treatment. (TIFF) [file pone.0161745.s004.tiff]

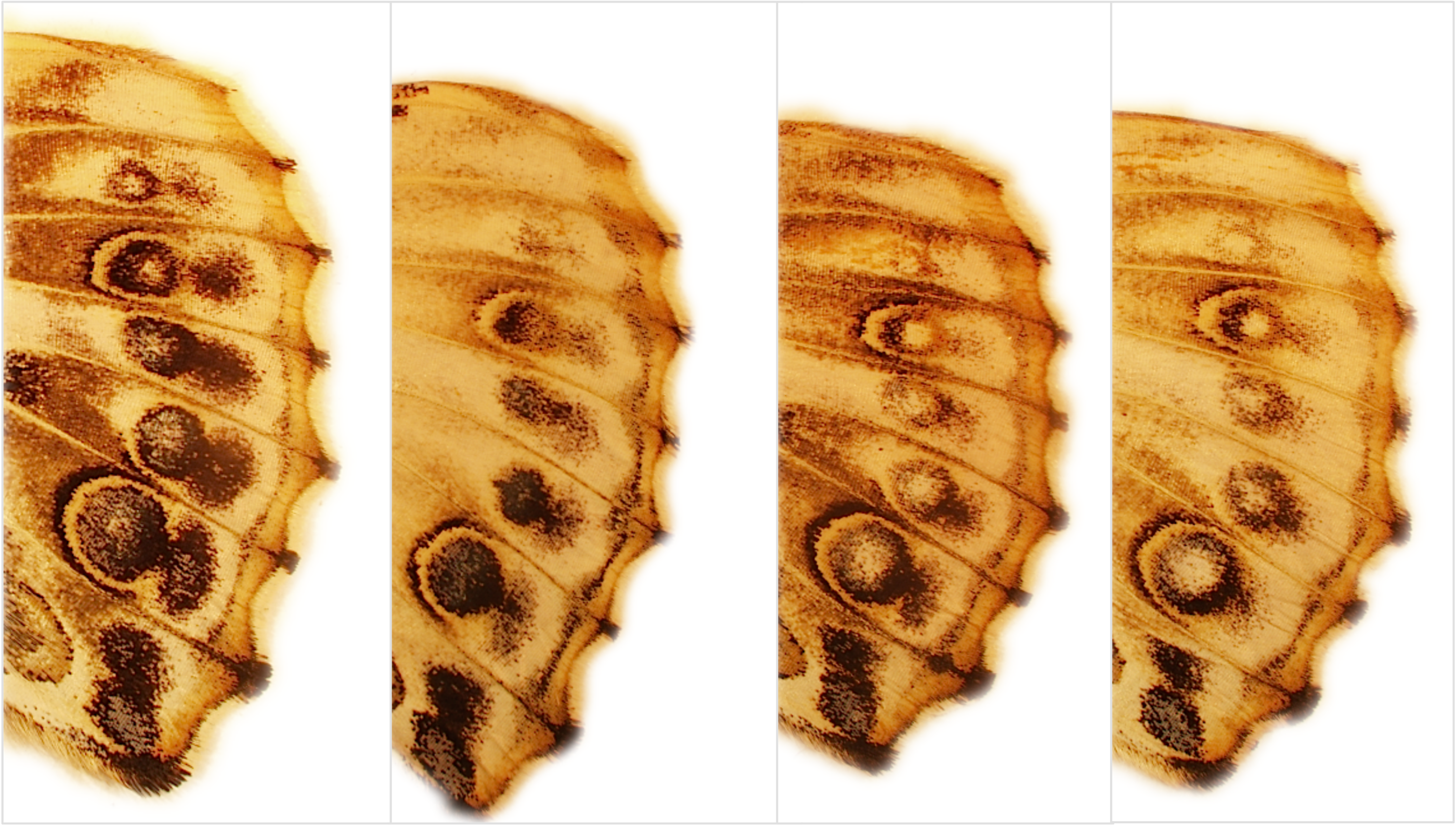

Supplement: S5 Fig — Butterflies exhibit a range of responses to heparin including complete loss of eyespots as shown in Fig 1, to bleaching and distortion of inner pattern elements with parafocal elements. A similar range of phenotypes is also observed following exposure to cold shock and sodium tungstate [29,40]. (TIFF) [file pone.0161745.s005.tiff]

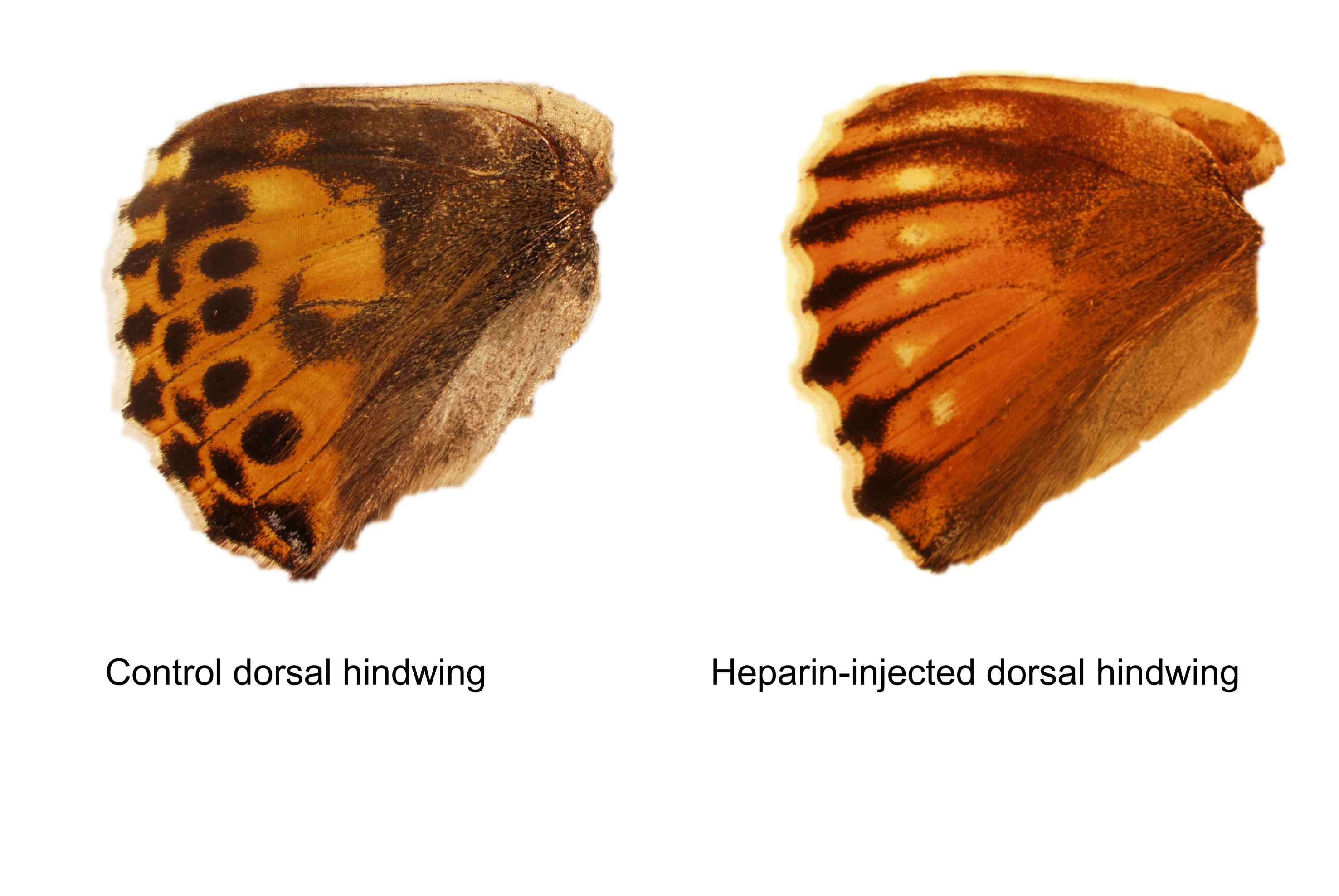

Supplement: S6 Fig — Heparin expands black pigmentation of parafocal elements and eliminates black pigment in dorsal spots. (TIFF) [file pone.0161745.s006.tiff]
